# VHL regulates the sensitivity of clear cell renal cell carcinoma to SIRT4-mediated metabolic stress via HIF-1α/HO-1 pathway

**DOI:** 10.1038/s41419-021-03901-7

**Published:** 2021-06-16

**Authors:** Ying Tong, Jinyan Kai, Shuo Wang, Yiwen Yu, Suhong Xie, Hui Zheng, Yanchun Wang, Yixuan Liu, Keyu Zhu, Xiaolin Guan, Lin Guo, Renquan Lu

**Affiliations:** 1 Department of Clinical Laboratory, Fudan University Shanghai Cancer Center, Shanghai, China; 2 Department of Oncology, Shanghai Medical College, Fudan University, Shanghai, China; 3 Department of Orthopaedics, Shanghai Jiaotong University Affiliated Sixth People’s Hospital, Shanghai, China

**Keywords:** Tumour-suppressor proteins, Renal cell carcinoma

## Abstract

Clear cell renal cell carcinomas (ccRCC) reprogram carbon metabolism responses to hypoxia, thereby promoting utilization of glutamine. Recently, sirtuin 4 (SIRT4), a novel molecular has turned out to be related to alternating glutamine metabolism and modulating the tumor microenvironment. However, the role of SIRT4 in ccRCC remains poorly understood. Here, we illustrated that the expression of SIRT4 is markedly reduced in cancerous tissues, and closely associated with malignancy stage, grade, and prognosis. In ccRCC cells, SIRT4 exerted its proapoptotic activity through enhancing intracellular reactive oxygen species (ROS). Heme oxygenase-1 (HO-1) is part of an endogenous defense system against oxidative stress. Nevertheless, overexpression of SIRT4 hindered the upregulation of HO-1 in von Hippel–Lindau (VHL)-proficient cells and repressed its expression in VHL-deficient cells. This discrepancy indicated that competent VHL withstands the inhibitory role of SIRT4 on HIF-1α/HO-1. Functionally, overexpression of HO-1 counteracted the promotional effects of SIRT4 on ROS accumulation and apoptosis. Mechanistically, SIRT4 modulates ROS and HO-1 expression via accommodating p38-MAPK phosphorylation. By contrast, downregulation of p38-MAPK by SB203580 decreased intracellular ROS level and enhanced the expression of HO-1. Collectively, this work revealed a potential role for SIRT4 in the stimulation of ROS and the modulation of apoptosis. SIRT4/HO-1 may act as a potential therapeutic target, especially in VHL-deficient ccRCCs.

## Introduction

Clear cell renal cell carcinoma (ccRCC), ranking as the most common subtype, accounts for ~70–75% of renal cell carcinomas (RCCs) [1]. It is known that von Hippel–Lindau (VHL) inactivation generally exists in ccRCC in the most common form, consequently leading to the activation of hypoxia inducible factor (HIF)-relevant hypoxia. Constitutive HIF activation primarily drives tumor progression and metastasis in renal carcinoma. As a hallmark of ccRCC, hypoxia signaling reprograms cancer cell metabolism to circumvent oxygen and nutrient shortages, as well as endows cancer cells with a proliferative advantage [2]. Meanwhile, ccRCC is associated with the reprogramming of metabolic pathways including oxygen sensing, metabolism of fatty acids, glucose, glutamine, and so on. Though previous investigations have made enormous improvements, the prognosis of ccRCC remains unfavorable due to its refractoriness to chemotherapy. As reported, RCC resistance could also be initiated through multiple mechanisms, such as the cellular microenvironment maladjustment and apoptotic death evasion [3]. Therefore, altering the hypoxic tumor microenvironment is a promising therapy for ccRCC.

Nowadays, the concept of “survival-associated pairwise gene expression states” has been generalized, whereby specific joint expression levels of a pair of genes as one entity is associated with cancer survival [4]. One approach for developing new therapy options in ccRCC would be to identify targets that functionally interact with VHL loss. Genes or proteins whose expressions are associated with lower tumor fitness and, consequently, better cancer patient survival in the context of VHL inactivation would theoretically be ideal targets for treating ccRCC.

Mitochondria protein sirtuin 4 (SIRT4) is a rather understudied member of the sirtuin family. One key function of SIRT4 is related to metabolism regulation. Glutamate dehydrogenase (GDH), which converts glutamate to α-ketoglutarate (αKG) in mitochondria, is regulated by ADP-ribosylation mediated by SIRT4 [5, 6]. Namely, SIRT4 is crucial for regulating mitochondria glutamine metabolism. Glutamine is critical for many fundamental functions in cancer cells, such as generating antioxidants to remove reactive oxygen species (ROS), maintaining mitochondrial metabolism, and activating cell signaling [7]. Thus, targeting glutamine metabolism may exhibit therapeutic potential to some extent. SIRT4 may have an enhanced role in the heart, kidneys, liver, and brain for its high expression [8]. A plethora of studies on SIRT4 in tumors demonstrated it as a tumor suppressor gene [9–11]. However, in other tumorous condition, SIRT4 may play an oncogenic role [12–14], which merits further studies in ccRCC for confirmation.

Hypoxia, a common condition in solid tumors, diminished apoptotic potential in carcinoma cells [15]. The apoptotic process of programmed cell death and its dysfunctions in malignancy has become the focus of extensive scientific research. Heme oxygenase-1 (HO-1) is a cytoprotective molecule with antioxidant, anti-inflammatory, and antiapoptotic properties. It promotes resistance in various stress-related conditions. In several studies, targeting HO-1 has been implemented as an antitumor therapy [16]. In addition, several signaling pathways such as phosphatidylinositol 3-kinase (PI3K/Akt) and p38-MAPK are involved in ROS generation and regulating HO-1 expression [17, 18], but the exact mechanisms in ccRCCs are incompletely understood.

Here, we demonstrated that SIRT4 was downregulated in ccRCC. Given that the mutation of VHL was a hallmark of ccRCC, we overexpressed SIRT4 in VHL-proficient and deficient cells, respectively, and conducted a comprehensive analysis of the relationship between SIRT4 expression and ROS roles in ccRCC cell lines. Meanwhile, a preliminary analysis of the relationship between SIRT4, HIF-1α, and HO-1 was performed as well. Thus, we provided a greater insight into mechanisms on the sensitivity of ccRCC cells to SIRT4- restricted glutamine metabolism, hence setting the foundation for further research on the VHL-deficient ccRCC patients.

## Material and methods

### Reagents and antibodies

2-Ketoglutaric acid (α-KG, CAS#328-50-7), EGCG (A606599), and N-acetyl-L-cysteine (NAC, CAS#616-91-1) were purchased from Sangon Biotech. BPTES (SML0601) and 2′,7′-dichlorofluorescein-diacetate (DCFH-DA, HY-D0940) were purchased from Sigma-Aldrich. DMEM was purchased from Hyclone. VH-298 (HY-100947) was purchased from MCE. The following antibodies were used for western blot: Sirt4 (sc-135797, Santa), HO-1 (sc-136960, Santa), HIF-1 alpha (PA1-184, Invitrogen), caspase-9 (CASP9) (sc-56076, Santa), Bax (50599-2-ig, Proteintech), Bcl-2 (12789-1-AP, Proteintech), and protein ladder (Thermo Fisher #26616).

### Tissue samples

ccRCC tissues and adjacent normal tissues were obtained from patients undergoing resection of renal cancer at the Department of Urology, Fudan University Shanghai Cancer Center. The final diagnosis of renal carcinoma was confirmed by histological analysis. Written informed consent was obtained from all participants.

### Cell culture

786-O cells and Caki-2 cells were cultured in 5% CO2 incubators, and maintained in Roswell Park Memorial Institute (RPMI-1640), containing 10% fetal bovine serum (Gibco) supplemented with penicillin–streptomycin (Invitrogen).

### Lentivirus-mediated overexpressing

Exogenous SIRT4, SIRT4-H161Y (an enzymatically inactive mutant of SIRT4), and HO-1 were overexpressed by the lentivirus. The pCDH-CMV-MCS-EF1-Puro plasmid was obtained from Systembio (SBI, Palo Alto, CA, USA). To produce lentiviruses, 3 × 10^4^ HEK293T cells per six-well plate well were cultured for 16–18 h till 80% confluency was reached. HEK293T cells were cotransfected by Lipofectamine 2000 (Invitrogen) mixed with plasmid SIRT4, sPAX2, and pMD2 in a ratio of 4:3:1. Following culture cells for an additional 48 h, the lentiviral supernatants were harvested and used in subsequent assays.

### TCGA database analysis

We downloaded the database of kidney renal clear carcinoma (KIRC) (TCGA, *Nature*, 2013) from cBioPortal. These samples were divided into two subclasses according to VHL status, analyzed relative gene expression was analyzed as well. Moreover, we acquired TCGA kidney renal clear carcinoma RNA-seq dataset from Cancer RNA-Seq Nexus (http://syslab4.nchu.edu.tw/), which included five subsets (stages I–IV and adjacent normals) and 601 samples, and consequently analyzed HO-1 expression in five subsets.

### Intracellular ROS level measurement

Intracellular ROS level was determined by staining of the cells with DCFH-DA. Cells were washed with PBS and incubated with 10 μM DCFH-DA for 30 min in a 37 °C incubator. Cells were processed using flow cytometry with a 485-nm excitation filter and a 538-nm emission filter.

### Western blot

Cells were lysed with the IP cell lysis buffer supplemented with 1 mM phenylmethanesulfonyl fluoride and 1 mM protease inhibitor cocktail. Western blot experiments were performed after the designated treatment and sample collection. Protein concentrations were determined by BCA assay before proteins were degenerated with loading. The cell lysate was separated by SDS-10% polyacrylamide gel electrophoresis and transferred to PVDF membranes (Invitrogen, ISEQ00010). After blocked with 10% nonfat powdered milk for 1.5 h at room temperature, membranes were incubated overnight at 4 °C with different primary antibodies. These membranes were incubated with HRP-conjugated secondary antibodies (1:5000). Signals were detected by the ECL chemiluminescence system (Amersham).

### Cell apoptosis

Cellular apoptosis was quantified by flow cytometry assay after annexin V–FITC and propidium iodide staining. According to instructions, cells were detached with 2.5% trypsin-EDTA and centrifuged at 1500 rpm for 5 min. The pellet was suspended in 300 µL of 1× binding buffer (FITC Annexin V Apoptosis Detection Kit I, BD Biosciences, USA). Flow cytometry and data analysis were conducted on a BD FACSCalibur.

### Clone formation assay

Caki-2 cells and 786-O cells, stably expressing SIRT4 or SIRT4-H161Y, as well as relative control cells were seeded into six-well plates at a density of 1000 cells per well. After cultivation with 1640 plus 10% serum for 10 days, all the cells mentioned above were fixed with 4% paraformaldehyde and stained with 1% crystal violet.

### Homology modeling and protein–protein docking

We downloaded the amino acid sequences of SIRT4 and HIF-1α from the UniProtKB database (http://www.uniprot.org/) to model the protein crystal structure. Homology modeling was applied with SWISS-MODEL (https://www.swissmodel.expasy.org/) to obtain the structure of SIRT4 and HIF-1α. PROCHECK was used to examine the stereochemical quality of the structure obtained from SWISS-MODEL in order to draw a Ramachandran plot. The interaction between SIRT4 and HIF-1α was simulated via ZDOCK Server (http://zdock.umassmed.edu/). Interaction interface residues between SIRT4 and HIF-1α were determined by PDBePISA (http://www.ebi.ac.uk/msd-srv/prot_int/pistart.html), with the default parameters. The protein–protein complex interactions were analyzed by PyMol.

### An outcome model for ccRCC

Univariate and multivariate Cox regression analysis was performed to identify the proper terms to build the nomogram. The forest was used to show the *P* value, HR, and 95% CI of each variable through “forestplot” R package. A nomogram was developed based on the results of multivariate Cox proportional hazards analysis to predict the 1-, 2-, 3-, and 5-year overall recurrence [19].

### Statistics

Statistical significance was determined using Student’s *t* test, Dunnett’s multiple comparisons test, and Sidak’s multiple comparisons test followed by GraphPad Prism 6 or SPSS software version 16.0 (SPSS Inc, Chicago, IL, USA). All data are presented as the mean ± standard deviation (SD) or mean ± standard error of the mean (SEM). *P* value < 0.05 was considered significant (*P < 0.05, **P < 0.01, ***P < 0.001, ns: no significance).

## Results

### ccRCC cells are sensitive to glutamine deprivation or inhibition

**Fig. 1:**
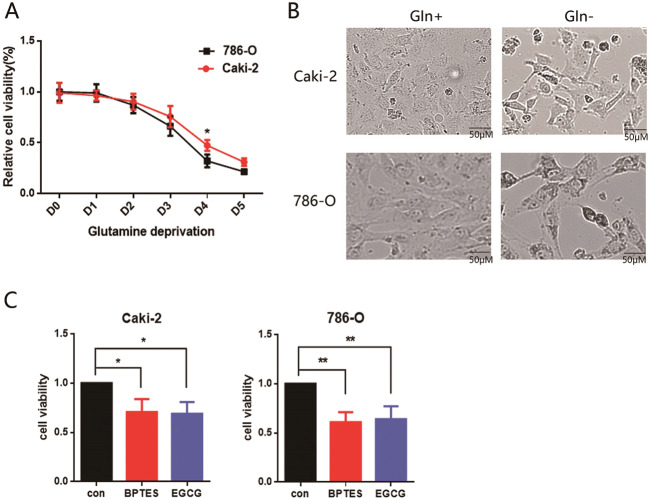
VHL-deficient cells more sensitive to glutamine metabolism. **A**, **B** VHL-proficient (Caki-2) and VHL-deficient (786-O) cells were cultured in complete medium or medium absence of glutamine. **A** Cell growth was determined by CCK8 and normalized to the corresponding cell type (Caki-2 or 786-O) grown in complete medium. **B** Representative phase-contrast photomicrograph was used to monitor cell survival after cultured for 96 h. **C** Caki-2 and 786-O cells were cultured for 48 h by complete medium with or without BPTES (10 μM)/EGCG (25 μM). Cell viability was determined by CCK8 assay. Error bars represent mean ± SD (*n* = 3). *P < 0.05, **P < 0.01. Student’s *t* test compared treated groups with corresponding control cells.

Nutrient availability is one of the key parameters for cells to make life-or-death decisions. Given the characteristic loss of the VHL functioning in ccRCC, we used VHL-defective (786-O) and VHL-proficient cells (Caki-2) to study the influence of glutamine metabolism on ccRCC cells. To recapitulate cell death under glutamine deprivation, we incubated 786-O and Caki-2 cells in a growth medium containing glucose but lacking glutamine. The viabilities of Caki-2 and 786-O cells upon glutamine deprivation were significantly lower than those in complete medium (Fig. 1A), and we confirmed the inhibition of viability by phase-contrast microscopy at 96 h after glutamine deprivation (Fig. 1B). Moreover, glutamine is converted into αKG through GDH or glutaminase (GLS). Thus, we furtherly cultured 786-O and Caki-2 cells with GLS inhibitor (BPTES) or GDH inhibitor (EGCG) for 48 h, then measured the cell viability. Notably, glutamine metabolism inhibited by BPTES or EGCG could suppress cell growth, and the effect was more pronounced in 786-O cells (Fig. 1C). These findings demonstrate that ccRCC cells with defective VHL are more sensitive to glutamine deprivation or inhibition.

### GDH inhibitor SIRT4 potentially serve as a diagnostic biomarker

**Fig. 2:**
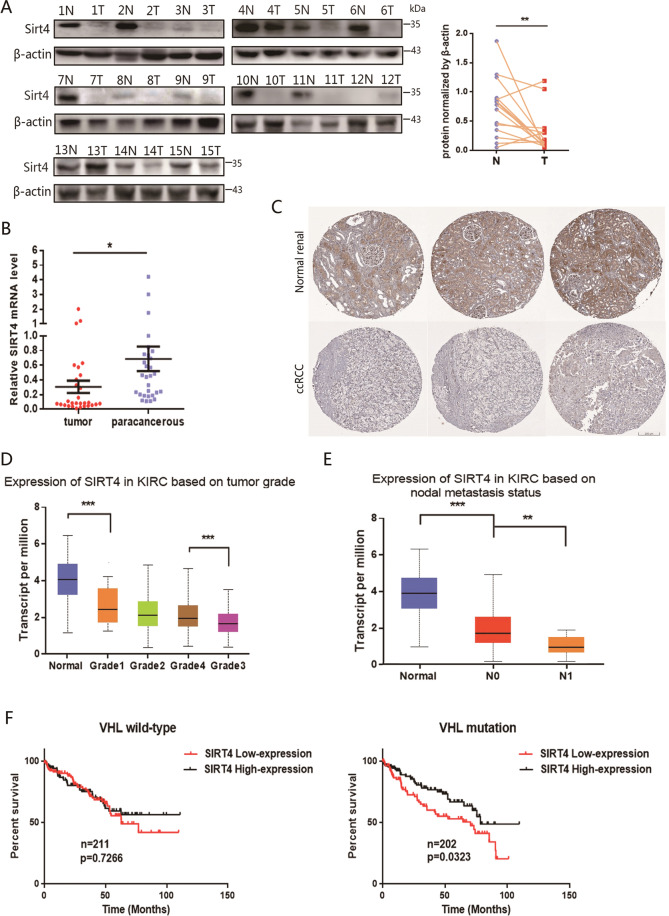
Reduced SIRT4 expression associated with malignancies stage, grade, and prognosis. **A** The protein expression levels of SIRT4 in ccRCC patients were validated by western blot (*n* = 15). β-actin serves as a loading control. A summary of the quantification analysis is shown on the right panel. **B** Relative mRNA expression of SIRT4 in ccRCC and normal tissues derived from our cohort (*n* = 29). Data were presented as means ± SEM. **C** The expression intensities of SIRT4 in normal renal tissues and renal carcinoma specimens were detected by IHC. Images were taken from the Human Protein Atlas (http://www.proteinatlas.org) online database. The relative expression of SIRT4 based on tumor grade (**D**) and nodal metastasis status (**E**) were analyzed via the TCGA RNA-seq database. **F** The survival analysis was carried out on a cohort of KIRC form the TCGA database (*Nature*, 2013). We classified the patients into two subgroups according to VHL status. Kaplan–Meier estimates of overall survival dependent on expression of SIRT4 (RNA-seq). Log-rank (Mantel–Cox) test was used to analyze OS. **P < 0.01, ***P < 0.001.

It has been demonstrated that cancer cells may exhibit metabolic dependencies distinguishing them from their normal counterparts [20]. Repression of vital metabolic enzymes in glutamine may provide novel therapeutic approaches to treat these refractory tumors. Given SIRT4, the mitochondrial-localized sirtuin that inhibits GDH, we investigated the expression of SIRT4 in tumor and paracancerous of ccRCC patients. Results indicated that both the protein and mRNA levels of SIRT4 in cancerous tissue (T) were significantly lower than their paired paracancerous tissue (N) (Fig. 2A, B). Besides, immunohistochemical evaluation also revealed lower expression intensity of SIRT4 in cancerous tissue than in normal renal tissues (Fig. 2C). In addition, SIRT4 might be closely involved in the process of ccRCC development and potentially serve as a diagnostic biomarker. Not surprisingly, lower SIRT4 levels were observed to be related to more advanced pathological grades (Fig. 2D) as well as more positive lymph nodes (Fig. 2E). Moreover, we classified the cohort of KIRC (TCGA, *Nature*, 2013) into VHL-WT and VHL-mutation subgroups. Further analysis revealed that high expression of SIRT4 possessed a significantly favorable prognosis in VHL-mutation patients with a profoundly improved overall survival (OS) (Fig. 2F). These data hinted that SIRT4 might have an advantageous, previously undetermined role in ccRCC and may provide novel therapeutic approaches to treat ccRCC, particularly in VHL-deficient patients.

### SIRT4 suppresses cell survival and induces ROS

**Fig. 3:**
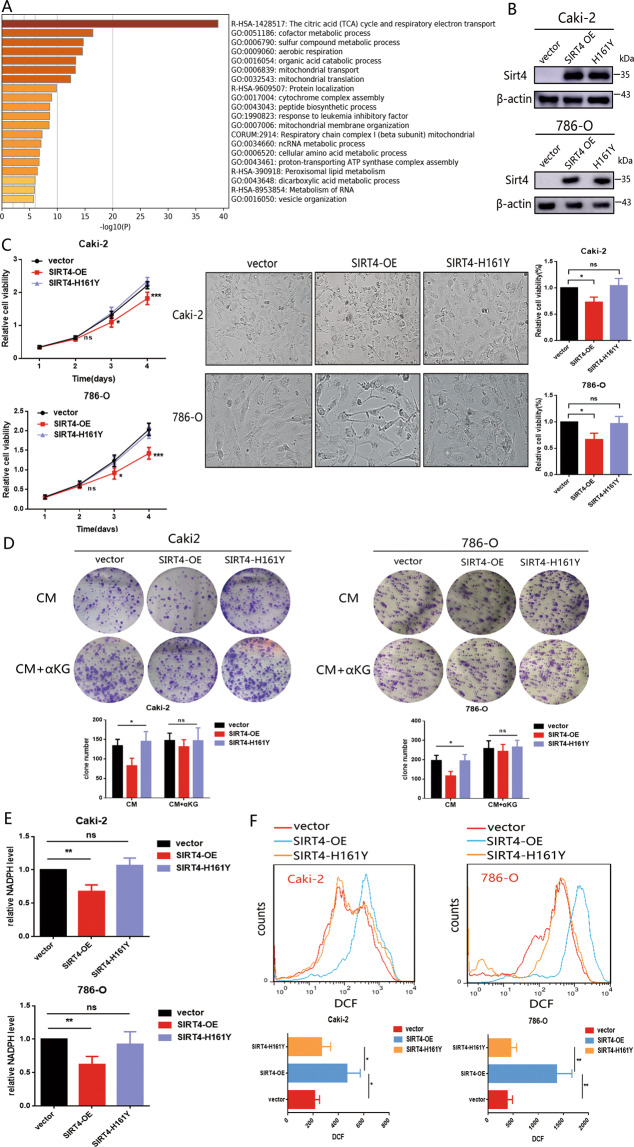
SIRT4-OE restrained cell survival and enhanced intracellular ROS. **A** KEGG pathway enrichment analysis based on SIRT4 association genes was performed by *Metascape*. **B** The efficiency of SIRT4 overexpression and SIRT4-H161Y reconstitution in Caki-2 and 786-O cells were validated by western blot. **C** Effects of SIRT4 or SIRT4-H161Y overexpression on cell growth was monitored by CCK8 (left panel) and representative phase-contrast photomicrograph (middle panel). The quantification analysis is shown as the right panel. **D** The effects of SIRT4-OE and H161Y reconstitution on colony formation capacity. Caki-2 and 786-O cells were cultured in a complete medium (CM) with or without supplemental α-KG (25 μM). A summary of the quantification analysis is shown underneath. **E** NADPH levels in cells with SIRT4 overexpression and H161Y reconstitution. **F** Intracellular ROS monitored by the DCFH-DA fluorescent probe (DCF) via FACS. Data are expressed as mean ± SD, experiments were done in three independent times. Throughout the figure, *P < 0.05, **P < 0.01, ns: no significance.

To better understand the mechanism of SIRT4, genes associated with SIRT4 in ccRCC were screened out by the Pearson correlation test via LinkedOmics (sub1). KEGG pathway enrichment of association genes (Pearson correlation > 0.3 or <−0.3) were enriched in the citric acid (TCA) cycle and respiratory electron transport (Fig. 3A). The data verified that SIRT4 acts as a metabolic regulator. Thus, we sought to investigate the effect of SIRT4 in ccRCC cells. Firstly, we overexpressed SIRT4 effectively in these cells by lentivirus (Fig. 3B). After culturing SIRT4 overexpressed (OE) and SIRT4 deficient (H161Y, catalytically inactive) cells in a complete medium, we noted that SIRT4-OE cells exhibited poor growth. And we further confirmed cell growth at day 3 by phase-contrast microscopy (Fig. 3C). Moreover, the colony formation assay revealed that overexpression of SIRT4 notably reduced cell survival. As reported, SIRT4 displays tumor suppressor activity through downregulating glutamine metabolism in several cancer types [21, 22], which deserves further probe in ccRCC cells. Thus, we cultured SIRT4-OE and SIRT4-H161Y cells in a complete medium with or without additional α-KG, which was a key metabolite of glutamine. Interestingly, exogenous α-KG rescued the clone formation capacity suppressed by overexpression of SIRT4, which furtherly validated that SIRT4 reduced cell utilization of glutamine for cell survival (Fig. 3D). In addition to energy-providing, glutamine carbons contribute to nicotinamide adenine nucleotide phosphate (NADPH) biosynthesis. Consistently, in SIRT4-OE cells, the NADPH level was sharply reduced (Fig. 3E). NADPH turned out to be an intracellular antioxidant that plays a significant role in cellular defense against oxidative stress. As a result, intracellular ROS was significantly increased in cells with SIRT4-OE rather than with H161Y reconstitution (Fig. 3F), highlighting that SIRT4 has the potential to induce ROS generation. Collectively, overexpression of SIRT4 remarkably restrained cell survival and altered tumor hypoxic microenvironment.

### SIRT4 sensitizes cell to apoptosis through intracellular ROS accumulation

**Fig. 4:**
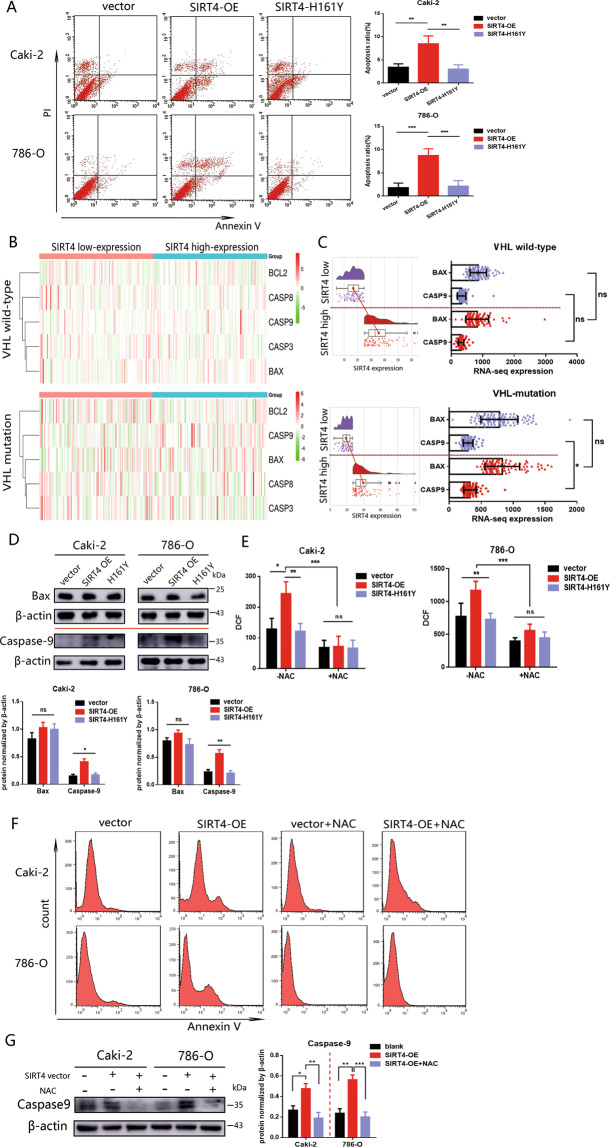
SIRT4-OE induced apoptosis by ROS enhancement. **A** The effect of SIRT4-OE and H161Y reconstitution on apoptosis was evaluated in Caki-2 and 786-O cells. Apoptosis (cells with annexin V positive) ratio was revealed as right. **B** Clustering heatmaps were performed based on the expression of SIRT4 and apoptosis-related genes (BCL2, CASP8, CASP9, CASP3, BAX) from the TCGA database. **C** Expression differences of CASP9 (caspase-9) and BAX between SIRT4-low and SIR4-high-expression subgroups based on the TCGA database (*Nature*, 2013). Data are plotted as means ± SD. **D** Protein expression levels of caspase-9 and Bax in Caki-2 and 786-O cells with SIRT4 overexpression or H161Y reconstitution. Densitometric analysis of western blots was exhibited underneath. After treating cells with NAC at a dose of 5 mM for 24 h, intracellular ROS levels were determined by DCFDA (DCF) staining (**E**), and apoptosis was determined by FACS (**F**). **G** Assess the effects of SIRT4 overexpression and NAC on protein caspase-9 expression by western blot. Densitometric analysis of caspase-9 is shown on the right panel. Throughout the figure, *P < 0.05, **P < 0.01, ***P < 0.001, ns: no significance.

The faithful execution of apoptosis is essential in avoiding multiple catastrophic disease states. Given the vital role of apoptosis induced by ROS [23, 24] in carcinoma, we assessed whether SIRT4 would sensitize cells to apoptosis via ROS accumulation. Indeed, overexpression of SIRT4 significantly elevated levels of cell apoptosis (Fig. 4A). Moreover, clustering heatmaps described the classification of SIRT4 expression and apoptosis-related genes. We noted that the level of CASP9 was significantly higher in SIRT4 high-expression group in VHL-mutation patients (Fig. 4B). Further analysis verified that in VHL-deficient ccRCC patients, the expression of CASP9 was substantially reduced in the SIRT4 low-expression subset compared to the SIRT4 high-expression subset. Nevertheless, in VHL wild-type ccRCC patients, CASP9 level has no significant difference between the two subsets (Fig. 4C). Indeed, the expression of CASP9 was significantly elevated in 786-O cells with SIRT4-OE (Fig. 4D). Further, to evaluate whether the apoptosis induced by SIRT4 was due to ROS overload, cells were cultured in the presence of N-acetylcysteine (NAC), a kind of ROS scavenger. Flow cytometry indicated that NAC simultaneously neutralized the intracellular ROS overload by SIRT4-OE (Fig. 4E) and reduced the apoptosis of SIRT4-OE cells (Fig. 4F). The addition of NAC significantly rescued the activation of CASP9 by SIRT4-OE (Fig. 4G). Collectively, these data suggested that in SIRT4-OE cells, apoptosis was induced by ROS accumulation.

### SIRT4 abrogates oxidative stress-induced HO-1 upregulation

**Fig. 5:**
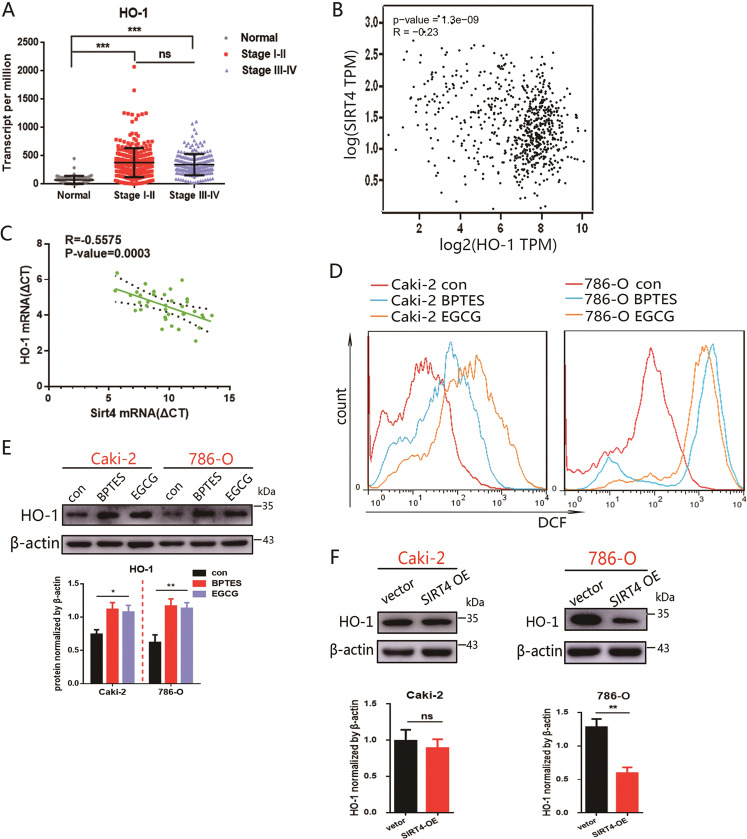
HO-1, stimulated by oxidative stress, the upregulation was intercepted by SIRT4-OE. **A** Relative expression of HO-1 in three subsets (stages I–IV and adjacent normal) were analyzed based on the database obtained from Cancer RNA-Seq Nexus. **B** Correlation between SIRT4 and HO-1 was analyzed by *GEPIA*. **C** Correlation between SIRT4 and HO-1 was validated based on RT-PCR results from twenty paired tissues of ccRCC patients. The lines of the 95% confidence bound for the linear regression are shown by black dashed lines. **D**, **E** Caki-2 and 786-O cells were treated with BPTES (10 μM) and EGCG (25 μM) for 48 h. **D** The intracellular ROS level was measured by DCFH-DA fluorescence. **E** The protein level of HO-1 was detected by western blot assay. The quantification of HO-1 is shown underneath. **F** The effect of SIRT4 overexpression on the protein expression level of HO-1 was disclosed by western blot. Densitometric analysis of HO-1 was exhibited underneath. **P < 0.01, ***P < 0.001, ns: no significance.

HO‐1 exerts antiapoptotic, antioxidant, and anti‐inflammatory effects [25, 26], its expression is upregulated by oncogenes [27]. Indeed, the RNA-seq dataset of ccRCCs from the TCGA indicated that the expression level of HO-1 was frequently lower in adjacent normal cases (*n* = 72) than ccRCC patients with stages I–IV (Fig. 5A). In addition, the results from the TCGA database and our cohort showed that HO-1 mRNA expression negatively correlated with SIRT4 (Fig. 5B, C). HO-1 is known to be stimulated by oxidative stress, serving both antioxidative and cytoprotective functions [28, 29]. As expected, cells treated with BPTES (a GLS1 inhibitor) and EGCG (a GDH inhibitor) exhibited the accumulation of ROS (Fig. 5D) and enhanced expression of HO-1 (Fig. 5E). These findings provoke our interest in the cytoprotective functions of HO-1 associated with oxidative stress. Interestingly, the expression of HO-1 was strongly and significantly inhibited by SIRT4-OE in 786-O cells but was scarcely changed in Caki-2 cells (Fig. 5F). Taken together, SIRT4 enhanced ROS generation, while refuses HO-1 upregulation, which was meaningful for modulating the tumor microenvironment.

### Competent VHL withstands the inhibitory role of SIRT4 on HIF-1α/HO-1

**Fig. 6:**
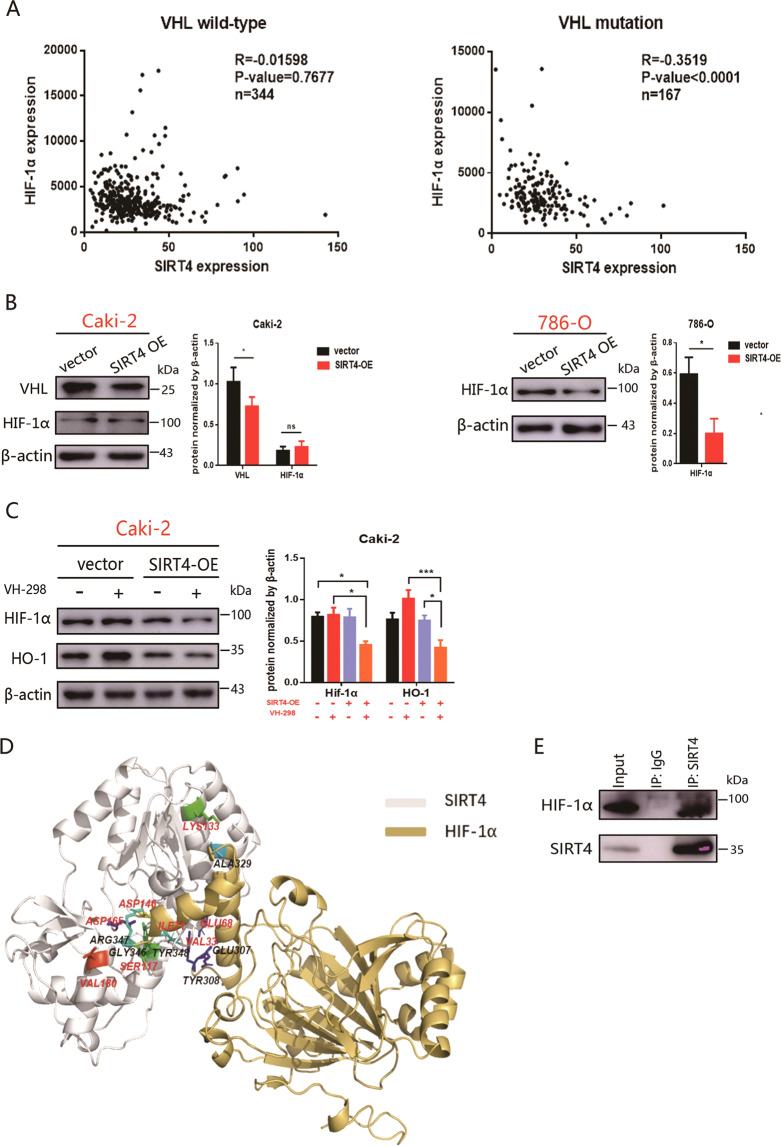
SIRT4 interacts with HIF-1α and directly suppresses the expression of HIF-1α. **A** Analyzed the correlation between HIF-1α and SIRT4 in mutated VHL and nonmutated VHL group from a TCGA cohort of ccRCC patients. **B** HIF-1α protein in SIRT4 overexpressed 786-O and Caki-2 cells were displayed by western blot. The quantification analysis is shown on the right panel. **C** Caki-2 cells were treated with VHL inhibitor, VH-298 (50 µM), for 24 h followed by western blot. Densitometric analyses of HIF-1α/HO-1 are shown on the right panel. **D** Molecular docking of SIRT4 and HIF-1α was realized by zdock. **E** The immunoprecipitation was used to analysis the interaction of exogenous SIRT4 with endogenous HIF-1α.

VHL status is an outstanding discrepancy between Caki-2 and 786-O cells. A principal role of VHL is in the regulation of HIF involved in oxygen sensing [30]. HO-1 is one of the downstream molecules of HIF-1α, and it is transcriptionally regulated by HIF-1α under hypoxia conditions [31]. Interestingly, HIF-1α was negatively correlated with SIRT4 in patients with VHL mutations and less pronounced in VHL wild-type patients (Fig. 6A). Moreover, in accordance with HO-1, the expression of HIF-1α in 786-O cells with SIRT4-OE was strongly reduced, while that in Caki-2 cells exhibited no significant difference (Fig. 6B). We next assessed whether the difference of HIF-1α expression in the SIRT4-OE subgroup of Caki-2 and 786-O cells was due to VHL status. To specifically rule out the role of VHL in HIF-1α, VH-298, a highly potent inhibitor of the VHL:HIF-α interaction, was used. As expected, in Caki-2 cells, HIF-1α/HO-1 was subsequently upregulated in the control group when treated with VH-298, while markedly reduced in the SIRT4-OE group (Fig. 6C). These data implied that competent VHL withstanding the inhibitory role of SIRT4 on HIF-1α and HIF-1α might be the intermediary molecule linking SIRT4-OE and HO-1 expression. Thus, we sought to investigate the mechanism by which SIRT4 depleted HIF-1α expression. Firstly, we modeled an optimal spatial structure of human SIRT4 and HIF-1α, and accomplished the density function energy requirements through the Ramachandran plot test (Fig. S1). Furtherly, we executed molecular docking between SIRT4 and HIF-1α via *zdock* (Fig. 6D). The binding sites of hydrogen bonds between SIRT4 and HIF-1α are shown in Table 1. Lastly, we verified the interaction of SIRT4 and HIF-1α. Given the low expression of SIRT4 in ccRCC cells, analyses were performed using Caki-2 cells with SIRT4 overexpression. Indeed, we noted exogenous SIRT4 to coimmunoprecipitate with endogenous HIF-1α (Fig. 6E). Thus, our data clearly illustrated that SIRT4 directly manipulates HIF-1α expression via protein–protein interaction.

**Table 1:** The binding sites of hydrogen bonds between SIRT4 and HIF-1α (proposed from the docking model).

Hydrogen bonds	SIRT4	Dist.[Å]	HIF-1α
1	VAL33	2.77	GLU307
2	ILE71	3.53	TYR308
3	ASP165	3.17	ARG347
4	ASP146	3.24	TYR348
5	GLU68	3.75	TYR308
6	LYS133	3.58	ALA329
7	VAL180	3.61	GLY346
8	SER117	2.46	TYR348

### HO-1 overexpression neutralizes ROS and blocks SIRT4-induced apoptosis

**Fig. 7:**
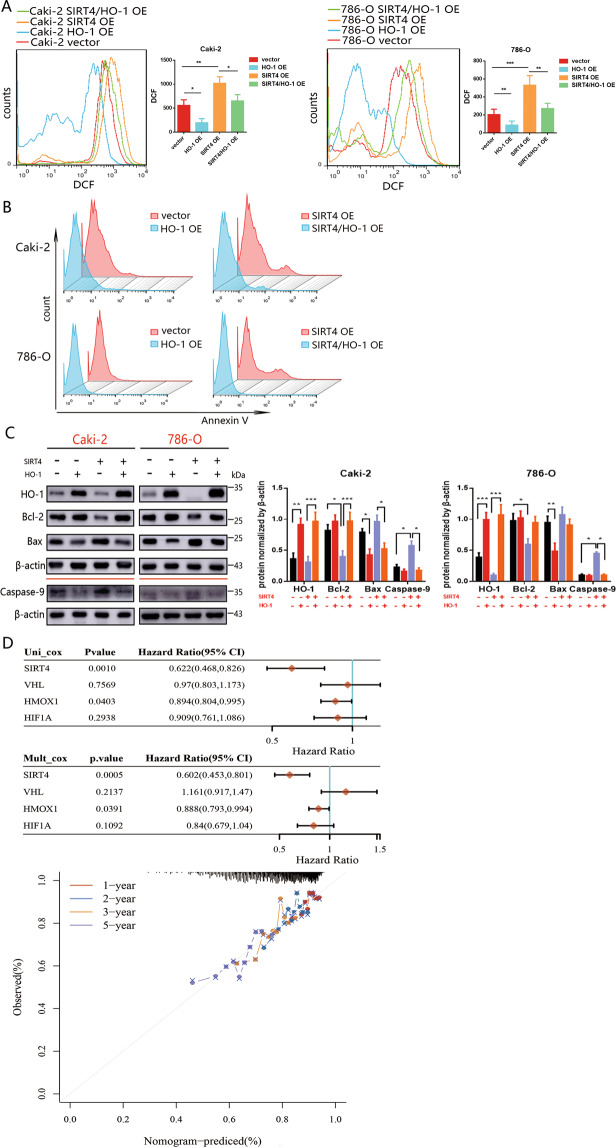
SIRT4 and HO-1 were significant effectors for ccRCC prognosis. **A** The effect of HO-1 overexpression on intracellular ROS levels in Caki-2 and 786-O cells were measured by DCFH-DA fluorescence. The quantification analysis (right panel) is shown. **B** The effect of HO-1 overexpression on apoptosis was analyzed by FACS. **C** Protein expression levels of apoptosis-associated genes after SIRT4 or HO-1 overexpression in 786-O and Caki-2 cells. The quantification analysis (right panel) is shown. **D** Hazard ratio and *P* value of constituents involved in univariate and multivariate Cox regression and some parameters of the SIRT4, VHL, HMOX1 (HO-1), and HIF1A (HIF-1α). Nomogram to predict the 1-, 2-, 3-, and 5-year overall survival of ccRCC cancer patients. A dashed diagonal line represents the ideal nomogram.

Given SIRT4 triggering apoptosis through ROS enhancement, we reasoned that restraining HO-1 upregulation was beneficial for maintaining adequate ROS. Indeed, overexpression of HO-1 counteracted the promotional effects of SIRT4 overexpression on ROS accumulation and apoptosis (Fig. 7A, B). We further confirmed the observations by evaluating apoptosis-related markers. Accordantly, overexpression of HO-1 elevated Bcl-2 level and reduced Bax and CASP9 expression (Fig. 7C). On the basis of our study, VHL, SIRT4, HIF-1α, and HO-1 were genes related to a few tumor-related biological processes, including metabolism, cancer development, and cellular stress response. We performed univariate and multivariate Cox regression analysis to investigate whether these elements were clinically independent prognostic factors for ccRCC patients. The results revealed that SIRT4 and HO-1 were the two independent prognostic factors. We further constructed a nomogram combining the two independent prognostic factors to provide an OS nomogram model to predict the probability of 1-, 2-, 3-, and 5‐year survival via a calibration curve (Fig. 7D). The analysis also demonstrated that SIRT4 and HO-1 were significant influence factors for ccRCC.

### SIRT4 modulates ROS level and HO-1 expression in a p38-MAPK depended way

**Fig. 8:**
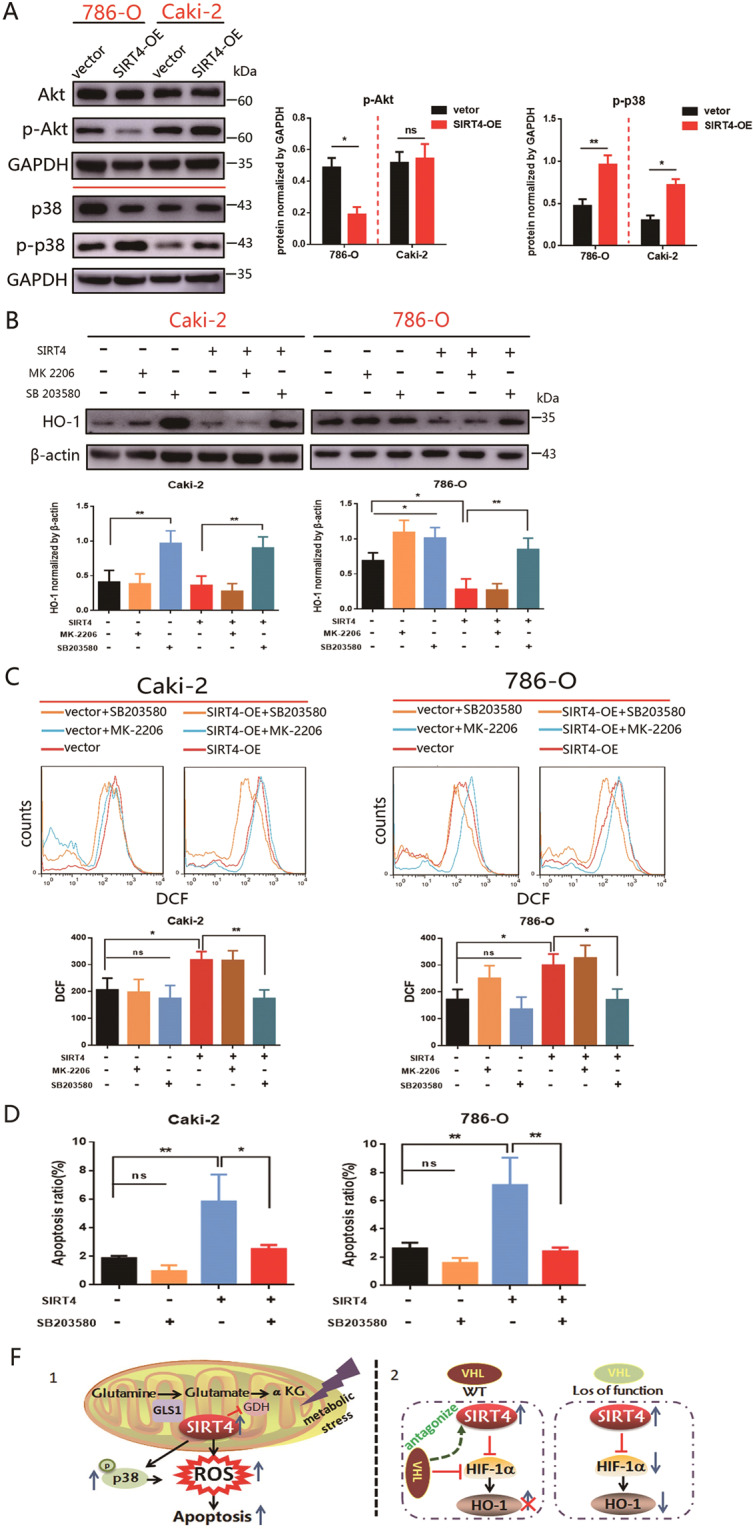
SIRT4-induced apoptosis in a p38-MAPK dependent manner. **A** Caki-2 and 786-O cells with SIRT4 overexpression were performed by western blot to analyze Akt or p38-MAPK pathway. Densitometric analysis was quantified on the right panel. **B** Cells with stable SIRT4 overexpression were treated by Akt (5 μM) or p38-MAPK antagonist (25 μM) for 24 h followed by western blot. Quantification of HO-1 is shown underneath. **C** Effects of Akt or p38-MAPK antagonist treatment on ROS generation in control and SIRT4 overexpression cells. A summary of the quantification analysis (below panel) is shown. **D** Effects of p38-MAPK antagonist treatment on apoptosis in control and SIRT4 overexpression cells. **E** A proposed model illustrating the distinct signaling components triggered by SIRT4. Error bars represent SD (*n* = 3). *P < 0.05, **P < 0.01, ns: no significance.

Akt and p38-MAPK pathways have been shown to play a significant role in regulating mitochondrial biogenesis, energy metabolism, and apoptosis [32, 33]. To further elucidate the possible mechanism associated with SIRT4-induced ROS accumulation and HO-1 astriction, the levels of phosphorylated (p) AKT and p-p38-MAPK were examined. As shown in Fig. 8A, p38 phosphorylation was significantly induced in SIRT4-OE cells compared to control cells. Akt phosphorylation was also greatly hindered by SIRT4 overexpression in 786-O cells. Furthermore, p38 inhibitor (SB203580) blocked the restriction role of SIRT4-OE on HO-1 upregulation both in Caki-2 and 786-O cells (Fig. 8B). Moreover, we have detected a significant reduction in intracellular ROS levels when treated with SB203580. By contrast, treatment with Akt inhibitor (MK-2206) did not result in an enhancement level of ROS (Fig. 8C). Lastly, our results manifested that cell apoptosis of SIRT4-OE was dramatically diminished when supplying the culture medium with SB203580 (Fig. 8D). Hence, our data demonstrated that SIRT4 might modulate ROS and HO-1 expression via accommodating p38-MAPK phosphorylation.

## Discussion

This study demonstrated that SIRT4 improved intracellular ROS and apoptosis in ccRCC cells through p38-MAPK phosphorylation. VHL regulates the sensitivity of ccRCC to SIRT4-mediated metabolic stress via the HIF-1α/HO-1 pathway. This study provided that SIRT4 repressed HIF-1α by interaction, and HIF-1α was the intermediary molecule linking SIRT4 and HO-1 expression. Moreover, a novel therapy was proposed wherein SIRT4/HO-1 acts as a potential therapeutic strategy, especially in VHL-deficient ccRCCs.

The stress-responsive mitochondrial sirtuin SIRT4 controls cellular energy metabolism in an NAD+-dependent manner and is implicated in cellular senescence and aging [34]. Relatively, SIRT4-mediated blockade of glutamine anaplerosis also could be a tumor suppressor mechanism [22, 35]. Here, we illustrated that the expression of SIRT4 reduced in cancerous tissues, and it was associated with malignancies stage and grade. These data were consistent with the observation that SIRT4 might be a novel biomarker for ccRCC [36, 37]. As reported, SIRT4 acts as a tumor suppressor by repressing proliferation, migration, and invasion ability of ccRCC cells [37]. SIRT4 also exerts a tumor-suppressive role by prompting abnormal cells to spontaneous death. Moreover, SIRT4 responds to metabolic stress by regulating mitochondrial ROS production. Whereas the association between SIRT4 and ROS was omnifarious in different diseases. A recent study indicated that SIRT4 promotes ROS generation [34, 38] and apoptosis [39]. However, in some cases, SIRT4 protects cells and tissue against damage by antiapoptosis [40] or preventing ROS [41]. Diverse roles of SIRT4 on apoptosis and ROS generation merits further studies in ccRCC for exploration. Here, we observed that overexpression of SIRT4-induced apoptosis via ROS accumulation and suppressed cell survival. VHL tumor suppressor gene inactivated in almost 80% of human ccRCCs, which leads to HIF-α protein stabilization and constitutive HIF activation [42–44]. Previous studies implicated that in RCC, cells deficient in the VHL gene took advantage of glutamine to generate citrate and lipids through reductive carboxylation of αKG [45]. These findings consolidated the exceptional value of VHL. In the current work, we reported that VHL-deficient ccRCC cells appeared to be more sensitive to glutamine inhibition or deprivation. Metabolic stress, characterized by nutrient, oxygen, and growth factor deprivation, prevalently exists in tumor microenvironments. Glutamine has a positive role in reducing oxidative stress damage [46]. Studies have suggested the induction of HO-1 occurs as an adaptive defensive mechanism, in response to oxidative stress and hypoxia [47]. High ROS is always accompanied by an increase of HO-1, whereas overexpression of SIRT4 obstructed the upregulation of HO-1, despite overloaded ROS. Also, HO‐1 exerts antiapoptotic, antioxidant effects by regulating antiapoptotic proteins [25, 48]. Consistently, overexpression of HO-1 in ccRCC cell lines counteracts ROS and apoptosis activated by SIRT4-OE. Reports have indicated that ROS regulates the stability and transcriptional activity of HIF-1α, which is a key regulator of HO-1 [49]. We indicated that HO-1 expression was positively correlated with HIF-1α and SIRT4 repressed HIF-1α by interaction. Thus, HIF-1α may be the intermediary molecule linking SIRT4 and HO-1 expression. Moreover, competent VHL withstands the inhibitory role of SIRT4 on HIF-1α/HO-1, which results in invariable expression of HIF-1α/HO-1 in Caki-2 cells. These observations highlighted novel strategies for the treatment of VHL-deficient tumors.

Metabolic stress induces cell death through ROS-induced apoptosis. Stress-activated MAPK pathways, such as p38-MAPK, seemed to be variable in different carcinomas but remained vital for the development and progression of cancer [50]. Recent studies have indicated that p38-MAPK signaling pathway controls adaptive responses to intracellular and extracellular stresses [51], and phosphorylation of p38-MAPK regulates the expression of apoptotic markers [52, 53]. Overexpression of SIRT4 engaged p38-MAPK phosphorylation for ROS enhancement and apoptosis. Conversely, the inhibitor of p38-MAPK (SB203580) counteracted the HO-1 suppression in the SIRT4-OE group.

The importance of our recent study consists in providing a novel mechanism for SIRT4 inducing ROS accumulation, as well as clarifying the regulatory modes between SIRT4 and HIF-1α/HO-1. Based on this mechanism, metabolism stress induced by SIRT4 is a potential therapeutic option for ccRCCs, especially VHL-deficient ccRCCs.

## Supplementary information

Sub 1

Figure S1
