# Supplementary figures and images for: VHL regulates the sensitivity of clear cell renal cell carcinoma to SIRT4-mediated metabolic stress via HIF-1α/HO-1 pathway
Source: Cell Death Dis. 2021 Jun 16;12(7):621. doi: 10.1038/s41419-021-03901-7 (PMC8209205; doi:10.1038/s41419-021-03901-7)

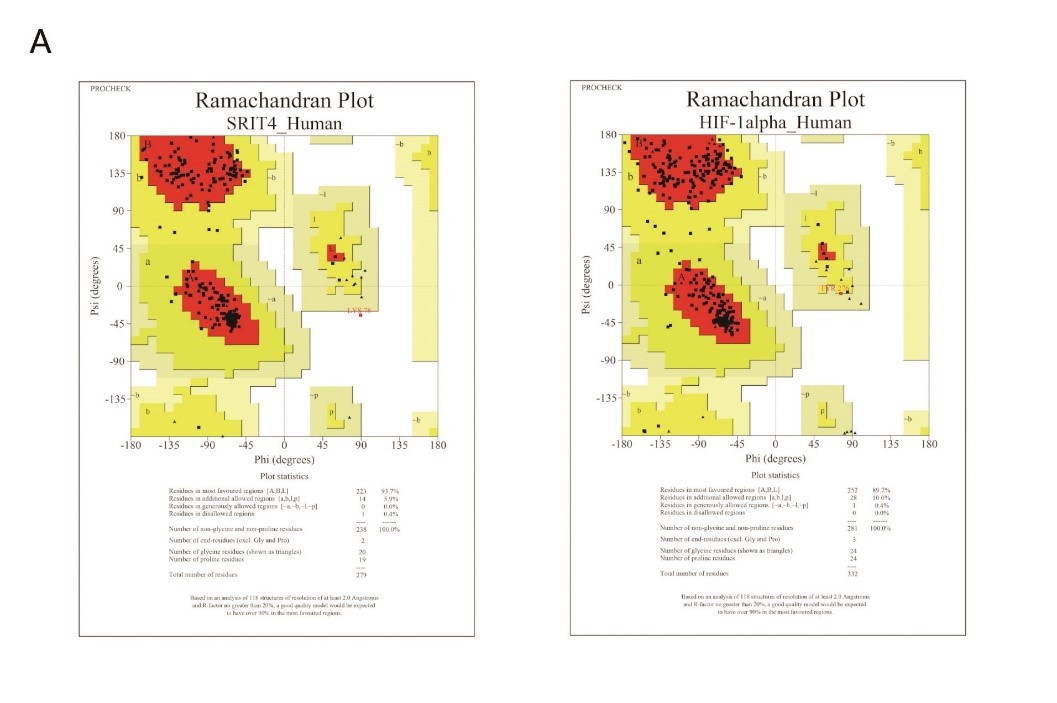

Supplement: Supplementary file 2 — Figure S1 [file 41419_2021_3901_MOESM2_ESM.jpg]
